# Dietary Index for Gut Microbiota and All-Cause Mortality in CKM Syndrome: Evidence of a J-Shaped Association and Partial Mediation by DII and CDAI

**DOI:** 10.2174/0118715303459900260505102519

**Published:** 2026-06-10

**Authors:** Xiaoqi Ma, Jingwen Zhang, Jinjin Huang, Ting Liu, Jingai Fang, Xiaodong Zhang

**Affiliations:** 1 Department of Nephrology, First School of Clinical Medicine, Shanxi Medical University, Taiyuan, China;; 2 Department of Nephrology, The First Hospital of Shanxi Medical University, Taiyuan, China

**Keywords:** CKM syndrome, dietary index for gut microbiota, dietary inflammatory index, composite dietary antioxidant index, all-cause mortality, cardiovascular mortality, national health and nutrition examination survey

## Abstract

**Introduction:**

Cardiovascular-kidney-metabolic (CKM) syndrome is a multisystem condition involving metabolic abnormalities and organ injury. While diet may influence health outcomes by modulating the gut microbiota, its association with mortality risk in patients with CKM syndrome remains unclear. This study aimed to examine the association between the Dietary Index for Gut Microbiota (DI-GM) and risks of all-cause and cardiovascular disease (CVD) mortality among individuals with CKM syndrome.

**Methods:**

This cohort study included 7,291 adults aged ≥ 20 years with CKM syndrome from NHANES 2009-2018. The DI-GM score (range 0-14; higher scores indicating a more favorable diet for beneficial microbiota) was derived from 14 dietary components and categorized into quartiles. Mortality outcomes were ascertained *via* linkage to the National Death Index. Multivariable Cox models were used to estimate Hazard Ratios (HRs), and restricted cubic splines examined dose-response relationships. Subgroup and mediation analyses were also conducted.

**Results:**

During follow-up, 546 all-cause and 166 CVD deaths were recorded. After adjustment, DI-GM showed a nonlinear association with all-cause mortality. Compared with the lowest quartile (Q1), the third quartile (Q3) had a significantly lower risk, whereas the highest quartile (Q4) showed no significant association. No significant link was observed between DI-GM and CVD mortality. Subgroup analyses indicated stronger protective effects in those with higher education, alcohol consumption, and without chronic kidney disease. Mediation analysis suggested that antioxidant and anti-inflammatory pathways partially explained the association.

**Discussion:**

DI-GM shows a J-shaped association with all-cause mortality in CKM syndrome, partially mediated by DII and CDAI, suggesting gut-inflammation pathways.

**Conclusion:**

Among patients with CKM syndrome, a moderate DI-GM score is associated with lower all-cause mortality risk, potentially mediated through anti-inflammatory and antioxidant mechanisms. DI-GM may help identify high-risk populations and inform dietary interventions.

## 1 INTRODUCTION

CKM syndrome, proposed by the American Heart Association, highlights the interrelatedness and mutual reinforcement among obesity, insulin resistance [1], chronic kidney disease [2, 3], atherosclerosis [4, 5], and cardiac dysfunction along a unified pathophysiologic axis [6-8]. Its staging framework centers on the accumulation of metabolic risk factors and the severity of cardiac and renal injury, reflecting a continuum from high risk to overt cardiovascular events [9]. Epidemiologic data indicate that the prevalence of CKM syndrome among US adults is as high as 89.4%, with more than 50% at stage 2 or higher, and that the disease burden showed no appreciable improvement from 2011 to 2020 [10]. These observations suggest that organ-specific approaches cannot account for the greater-than-additive accumulation of risk, highlighting the need to identify modifiable pathways at the systems level.

The gut microbiota has emerged as a key hub linking the metabolic, renal, and cardiovascular axes [11, 12]. The microbiota and its metabolites, such as Short-Chain Fatty Acids (SCFAs), trimethylamine N-oxide (TMAO), and lipopolysaccharide (LPS), may influence blood pressure regulation [13-15], atherosclerosis progression [16, 17], and renal fibrosis [13, 18, 19] by modulating energy metabolism [20, 21], lipid metabolism, endothelial function, and low-grade inflammation [22-24]. Persistent low-grade inflammation and oxidative stress have repeatedly been implicated in CKM-related adverse outcomes [25]. As a malleable upstream factor, diet can reshape microbiome diversity and the metabolomic profile, thereby acting on these pathways [26-28]. Accordingly, evaluating modifiable dietary patterns along the diet-microbiota-host metabolism/inflammation axis has theoretical and practical value for risk stratification and intervention in CKM populations [29].

The DI-GM was developed based on systematic reviews of evidence linking adult diets to the microbiota and integrates 14 food and nutrient indicators associated with a healthy microbiome profile [30]. Beneficial components (eg, fermented dairy products, whole grains, dietary fiber, and selected fruits and vegetables) and unfavorable components (eg, red/processed meats, refined grains, and high-fat diets) are weighted to quantify how microbiota-friendly an individual’s diet is. DI-GM has been associated with multiple populations and outcomes, including metabolic dysregulation, hepatic and renal function, inflammatory phenotypes, and cardiovascular risk phenotypes, suggesting that it may reflect a microbiota-oriented dietary pattern beneficial to the host, potentially by attenuating inflammation and enhancing antioxidant capacity [31]. However, prospective evidence regarding mortality outcomes among individuals with CKM syndrome is lacking, particularly concerning dose-response relationships with all-cause and cardiovascular mortality and potential mediating pathways.

Against this background, we constructed a prospective cohort using NHANES 2009-2018, enrolling Variable Definitions and Measurements 7,291 participants aged 20 years or older with CKM stages 1-4. DI-GM was calculated from 24-hour dietary recall data, and all-cause and cardiovascular mortality through December 31, 2019, were ascertained by linkage to the National Death Index. We evaluated associations between DI-GM and mortality using multivariable Cox proportional hazards models, characterized non-linear dose-response relationships with restricted cubic splines, and conducted subgroup and interaction analyses. In addition, mediation analyses incorporating the Composite Dietary Antioxidant Index (CDAI) and the Dietary Inflammatory Index (DII) were used to explore potential inflammatory and antioxidant pathways linking DI-GM to mortality [32, 33]. We hypothesized that more favorable DI-GM levels would be associated with a lower risk of all-cause mortality and that anti-inflammatory and antioxidant mechanisms would partially mediate this protective association. This study may provide actionable risk markers and mechanistic insights to inform dietary assessment and precision nutrition interventions for CKM populations.

## 2 MATERIALS AND METHODS

### 2.1 Study Design and Data Source

This cohort study used data from the NHANES [34], which employs a complex, multistage, stratified probability sampling design to represent the health and nutritional status of the noninstitutionalized civilian US population. NHANES protocols were approved by the National Center for Health Statistics (NCHS) Research Ethics Review Board, and written informed consent was obtained from all participants. Survey design, sampling methods, and detailed documentation are available at the NHANES website (https://www.cdc.gov/nchs/nhanes/).

We combined data from five consecutive 2-year cycles (2009-2018). Participants were eligible if they were aged 20 years or older and had complete data required to stage CKM syndrome. Exclusions were age <20 years or pregnancy; missing DI-GM exposure; missing key variables for CKM staging; missing follow-up information from linkage to the National Death Index (NDI); missing data on key covariates; and physiologically implausible energy intake (<800 or >8000 kcal/day). The final analytic sample included 7,291 participants; the selection process is shown in Fig. (**1**). The final analytic sample included 7,291 participants, including both male and female individuals; the selection process is shown in Fig. (**1**).

### 2.2 Variable Definitions and Measurements

#### 2.2.1 CKM Syndrome Staging

CKM staging followed the latest framework proposed by the American Heart Association (Supplementary Table **S2**) [35], which integrates indicators from multiple domains, including metabolic risk factors, kidney disease, and CVD (Supplementary Table **S1**) [6]. Staging incorporated Body Mass Index (BMI), blood pressure, blood glucose, blood lipids, urine albumin-to-creatinine ratio, and estimated glomerular filtration rate [36]. CKD staging followed the KDIGO clinical practice guideline.

#### 2.2.2 DI-GM

The Digestible Intestinal-Gut Microbiota (DI-GM) is a composite score that reflects the relationship between diet and the gut microbiota, comprising 14 food groups or nutrients. In accordance with the criteria established by Kase *et al.*, we classify these 14 items, avocado, broccoli, chickpeas, coffee, cranberries, fermented dairy products, dietary fiber, green tea (not detailed in NHANES), soybeans, and whole grains as positive factors; red meat, processed meat, refined grains, and high-fat diets (defined as fat contributing ≥40% of total energy) as negative components. Consequently, the DI-GM employs a binary scoring rule: a score of 1 is assigned to beneficial components when intake is at least the sex-specific median, and 0 otherwise; for unfavorable components, a score of 1 is assigned when intake is below the sex-specific median, and 0 otherwise [30] (Supplementary Table **S3**).

For analysis, we conducted a visual assessment of the DI-GM score distribution. Based on the observed pattern, we categorized total DI-GM scores into four groups: Q1 (1-4, n = 2,913), Q2 (5, n = 1,660), Q3 (6, n = 1,354), and Q4 (7-14, n = 1,364), with Q1 serving as the reference. This grouping method was chosen based on equal group size and the robustness of subsequent between-group comparisons. This classification approach is consistent with the grouping strategy used in prior DI-GM studies [37] (Supplementary Figure **S2**).

#### 2.2.3 CDAI and DII

The CDAI and the DII are instruments for evaluating dietary inflammation. The CDAI quantifies the combined intake of dietary zinc, selenium, vitamins A, C, and E, and carotenoids, thereby capturing cumulative antioxidant effects more comprehensively than single-nutrient assessments [38, 39]. These components were derived from two nonconsecutive 24-hour dietary recalls in NHANES. The CDAI is calculated by summing the standardized intakes for each antioxidant (each intake minus the sex-specific mean, divided by the sex-specific standard deviation). The formula is: CDAI = ∑ (each intake - mean value) / SD (n = 6).

As we know, the DII incorporates six inflammatory markers: interleukin-1β (IL-1β), interleukin-4 (IL-4), interleukin-6 (IL-6), interleukin-10 (IL-10), tumor necrosis factor-α (TNF-α), and C-Reactive Protein (CRP) [40, 41]. The DII assesses the inflammatory potential of diets by integrating information from 45 pro- and anti-inflammatory food parameters [42]. We computed the composite DII (C-DII) scores using NHANES 24-hour dietary recall data. In NHANES [43], 28 food parameters were available for DII calculation [44]. Nutrient- and energy-adjusted DII scores per 1000 kilocalories were calculated using energy-density methods, based on standardized global intakes from 11 populations worldwide.

### 2.3 Outcomes

Primary outcomes were all-cause mortality and CVD mortality. CVD mortality included deaths due to heart disease (ICD-10 codes I00-I09, I11, I13, I20-I51) and cerebrovascular disease (I60-I69). Mortality was ascertained by probabilistic linkage to the NDI with follow-up through December 31, 2019. In total, 546 all-cause deaths occurred, including 166 CVD deaths.

### 2.4 Covariates

Based on prior literature and biological plausibility, covariates included demographic and socioeconomic characteristics (age, sex, race, education, and family poverty-to-income ratio (PIR)); Anthropometrics (BMI and waist circumference); Lifestyle factors (smoking status (never, former, current) and alcohol use (never, former, current)); Clinical biomarkers (lipid profile (total cholesterol, triglycerides, HDL-C, LDL-C), kidney function indicators (uric acid, blood urea nitrogen, creatinine), glycemic parameters (fasting plasma glucose, hemoglobin A1c), and urine albumin-to-creatinine ratio); Dietary intake (total energy, total fat, and dietary cholesterol from 24-hour dietary recall(s)); and Medical history (hypertension, diabetes, chronic kidney disease, metabolic syndrome, malignancy, and CVD).

### 2.5 Statistical Analysis

Given NHANES’s complex sampling design, all analyses incorporated survey weights, using the 2-day dietary recall weight (WTDR2D) to enhance representativeness for the target population. Categorical variables were summarized as weighted percentages, and continuous variables as weighted means with standard errors or medians with interquartile ranges, according to distributional characteristics.

Primary analyses used a complete-case approach. Because the proportion of missingness in the primary outcome (mortality) exceeded 30% (Supplementary Figure **S1**), participants with missing mortality data were excluded. Missingness in other covariates was handled according to prespecified exclusion rules to maximize the completeness and comparability of the analytic sample. Multicollinearity was assessed using the Variance Inflation Factor (VIF), and only variables with VIF <5 were retained in multivariable models (Supplementary Table **S4**).

Cox proportional hazards models were used to estimate associations between the DI-GM score and all-cause and cardiovascular mortality. The DI-GM score was categorized into four groups (Q1: 1-4; Q2: 5; Q3: 6; Q4: 7-14), with Q1 as the reference. We reported Hazard Ratios (HRs) and 95% Confidence Intervals (CIs) for each category and tested for a linear trend. We specified three progressively adjusted models: Model 1 (age, sex, and race); Model 2 (Model 1 plus education, PIR, BMI, smoking status, and alcohol use); Model 3 (Model 2 plus hypertension, diabetes, chronic kidney disease (CKD), malignancy, metabolic syndrome, total energy intake, and dietary cholesterol intake).

To examine potential non-linear associations between DI-GM and mortality, we fitted Restricted Cubic Spline models (RCS) with 4 knots, adjusted for age, sex, and race. Nonlinearity was evaluated with likelihood ratio tests, with *p* < 0.05 indicating statistical significance.

Kaplan-Meier curves were used to depict survival across DI-GM categories, and differences were compared using the log-rank test. In the RCS and Kaplan-Meier curves, we used Model 1, which adjusted for age, sex, and race. This approach allows for the visualization of the fundamental dose-response shape and survival patterns while minimizing potential bias associated with the over-adjustment of variables. Furthermore, it ensures that the intrinsic non-linear relationship between the DI-GM and mortality is captured with maximum statistical power prior to the inclusion of complex clinical confounders.

Subgroup analyses stratified by age, sex, race, education, PIR, BMI, smoking, alcohol use, comorbidities (hypertension, diabetes, CKD, CVD, malignancy, metabolic syndrome), and CKM stage were conducted; interactions were evaluated with likelihood ratio tests, and results were displayed as forest plots.

Mediation analyses evaluated whether the CDAI, DII, and BMI mediated the association between DI-GM and mortality. Using the R package mediation, indirect effects and 95% CIs were estimated with 1000 bootstrap resamples.

Our study treated DI-GM as a continuous variable in a sensitivity analysis assessing its association with all-cause and cardiovascular mortality risk among patients with CKM. All analyses were conducted in R version 4.3.2 using the survey and survival packages for complex survey analyses. All hypothesis tests were two-sided, and *p* < 0.05 was considered statistically significant.

## 3 RESULTS

### 3.1 Characteristics of the Study Population

A total of 7,291 participants with CKM syndrome were included in the study. As summarized in Table **1**, patient characteristics varied markedly with advancing CKM stage. Individuals in CKM-4 were older (median age, 66 years; IQR, 58.0-74.0), had a higher proportion of men (53.4%), and had lower educational attainment and lower income-to-poverty ratios. Metabolic profiles worsened with higher CKM stage. BMI increased from 27.0kg/m2 in CKM-1 to 30.0kg/ m2 in CKM-3 (*p* < 0.001). Glycemic and renal markers also deteriorated in a stage-dependent fashion: fasting glucose rose from 98 mg/dL to 107 mg/dL, serum creatinine from 0.83 mg/dL to 0.96 mg/dL, and serum uric acid from 5.03 mg/dL to 5.80 mg/dL (all *p* < 0.001). Participants in CKM-4 had the highest smoking prevalence (60.7%) but the lowest mean daily energy intake (1,870 *vs* 2,083 kcal; *p*<0.001; CKM-4 *vs* CKM-1). The DII increased across CKM stages (1.08 to 1.55; *p* < 0.001), whereas the DI-GM score did not differ significantly by stage (*p* = 0.08). During the median follow-up, 546 all-cause deaths (5.6%) occurred, including 166 cardiovascular deaths (29.0%). The proportion of all-cause deaths increased from 1.4% in CKM-1 to 17.0% in CKM-4 (*p* < 0.001), and CKM-4 had the highest proportion of cardiovascular deaths (35%).

### 3.2 COX Model Between DI-GM and Mortality

In Cox proportional hazards models (Fig. **2**, Supplementary Table **S5**), after adjustment for age, sex, and race, higher DI-GM categories were inversely associated with all-cause mortality. Compared with Q1, the HR was 0.62 (95% CI: 0.46-0.83; *p*=0.001) for Q4 and 0.57 (95% CI, 0.39-0.84; *p*=0.005) for Q3. After further adjustment for socioeconomic, lifestyle, and clinical factors, the associations attenuated: for Q4, the HR was 0.78 (95% CI, 0.57-1.07; *p* = 0.13); only Q3 remained statistically significant (HR, 0.67; 95% CI, 0.46-0.98; *p* = 0.04). For cardiovascular mortality, no significant association with DI-GM was observed in any model (Model 3: Q4 *vs* Q1, HR, 0.95; 95% CI, 0.49-1.85; *p*>0.90).

### 3.3 Dose-response Relationship

Restricted cubic spline analyses (Fig. **3**) indicated a nonlinear association between the DI-GM score and all-cause mortality (*p* for nonlinearity = 0.001). Below a DI-GM score of 5.0, mortality risk increased as the score decreased (at DI-GM = 1.0, HR: 1.46; 95%CI: 1.01-2.11). Above 5.0, the association was protective; at DI-GM = 10.0, risk was 39% lower (HR: 0.61; 95%CI: 0.37-1.00). For cardiovascular mortality, a nonlinear pattern was detected (*p* for nonlinearity < 0.001), but the overall association was not statistically significant (χ^2^ = 0.50; *p* = 0.780). The HRs at DI-GM were 1.26 (95% CI: 0.66-2.37) and 1.21 (95% CI: 0.49-2.98) for HRs of 1.0 and 10.0, respectively.

### 3.4 Survival Analysis

As shown in the Kaplan-Meier curve (Fig. **4**), there were significant differences in all-cause survival (log-rank *p* = 0.041). After a follow-up period of 100 months, Q4 had a 3.3% survival advantage over Q2; at 125 months, Q3 had the greatest survival advantage, consistent with the RCS results. In terms of cardiovascular mortality, the survival curves did not differ significantly across DI-GM categories (log-rank *p* = 0.75).

### 3.5 Subgroup Analyses

The inverse association between DI-GM and all-cause mortality was more pronounced in several subgroups (Fig. **5**, Supplementary Table **S6**). Among individuals with higher education, the risk was 12% lower (HR: 0.88; 95%CI: 0.81-0.95; *p* = 0.001; interaction *p* = 0.005). Among drinkers, risk was 10% lower (HR: 0.90; 95%CI: 0.85-0.96; *p*=0.001), whereas no association was observed among nondrinkers (interaction *p*=0.038). Among those without CKD, risk was 11% lower (HR: 0.89; 95%CI: 0.83-0.96; *p*=0.002), whereas no significant protective association was observed among those with CKD (interaction *p*=0.022). Significant benefits were also observed among participants aged <60 years, non-Hispanic Black participants, and those with BMI <25kg/m^2^. We identified no significant associations between DI-GM and cardiovascular mortality in any subgroup (Fig. **6**).

### 3.6 Mediation Analysis

Each 1-unit increase in the DI-GM score was associated with an absolute reduction of 0.52% in all-cause mortality risk (β = −0.0052; 95%CI: −0.0084 to −0.0016; *p* < 0.001). The CDAI mediated 17.9% of the association (average causal mediation effect (ACME): −0.0009; 95%CI: −0.0016 to −0.0002; *p* = 0.012), and the DII mediated 27.6% (ACME: −0.0014; 95%CI: −0.0027 to −0.0001; *p* = 0.038). For cardiovascular mortality, neither the total effect nor the mediation effects were statistically significant (Fig. **7**, Supplementary Table **S7**).

### 3.7 Sensitivity Analysis

Our analysis treating DI-GM as a continuous variable revealed a weak inverse association with all-cause mortality in minimally adjusted models, which was attenuated to non-significance after full adjustment (Model 3: HR 0.96, 95% CI 0.90-1.03, *p*=0.2). No significant association was observed between DI-GM and CVD mortality across any of the models (Supplementary Table **S8**). These results are consistent with the findings presented earlier in the text.

## 4 DISCUSSION

Using NHANES 2009-2018 data with linked mortality follow-up, we evaluated the association of the DI-GM with all-cause and cardiovascular mortality among adults with CKM syndrome. After multivariable adjustment, a moderate DI-GM level (Q3, score 6) was associated with a 33% lower risk of all-cause mortality compared with the lowest quartile (Q1, score 1-4; HR: 0.67; 95%CI: 0.46-0.98). Restricted cubic spline analysis revealed a J-shaped dose-response curve, with the risk nadir occurring at scores 5-7. In mediation analyses, the CDAI and the DII accounted for 17.9% and 27.6% of the association, respectively, which suggested partial mediation through antioxidant and anti-inflammatory pathways. No statistically significant association was observed between DI-GM and cardiovascular mortality in the overall cohort.

Our findings align with those of Zheng *et al.* [45], who used NHANES 2007-2018 data, reported lower all-cause mortality among individuals with metabolic syndrome who had higher DI-GM (HR: 0.72; 95% CI: 0.61-0.85). Both studies employed multivariable Cox proportional hazards models; our analysis focused on the CKM population, in which the pathophysiology is more complex and better reflects the multi-organ interactions among nutrition, the gut microbiome, and the host. In contrast, a cross-sectional study by Niu *et al.* [46] reported an inverse association between DI-GM and prevalent metabolic syndrome (OR: 0.84; 95%CI: 0.70-1.01). These differences likely reflect study design: cross-sectional analyses address disease prevalence, whereas our mortality-linked approach evaluates fatal endpoints. In advanced CKM (*e.g*., eGFR <30 mL/min/1.73 m^2^), a heightened inflammatory burden may attenuate the apparent protection of higher DI-GM, consistent with the risk rebound at higher scores on the J-shaped curve.

Mechanistically, DI-GM may influence outcomes in CKM through several pathways. Diets rich in fiber and prebiotics increase SCFA production [47, 48], suppress the activation of the NF-κB signaling pathway [49-51], and reduce proinflammatory cytokines such as interleukin 6 (IL-6) and Tumor Necrosis Factor α (TNF-α) [52], consistent with the mediation observed for CDAI [53]. DI-GM-related dietary patterns may also modulate gut microbiota composition [54, 55], improve insulin sensitivity [56, 57], and reduce ectopic fat deposition, aligning with the mediating role suggested by DII [58]. There is evidence that the accumulation of uremic toxins threatens intestinal barrier integrity and reduces SCFA synthesis by approximately 50% in CKD stages 4-5 [59, 60], which could contribute to immunometabolic uncoupling and attenuate protection in subgroups with high DI-GM scores. As an illustrative example, the Mediterranean diet, characterized by abundant fruits, vegetables, olive oil, and fish, tends to yield higher DI-GM scores and provides ≥14 grams of dietary fiber per 1,000 kcal, including Microbiota-Accessible Carbohydrates (MACs) [61, 62]. Researchers have found that MACs promote SCFA-producing microbial taxa, including butyrate producers, thereby enhancing immune function and improving glucose-lipid metabolism and blood pressure regulation, ultimately reducing cardiovascular risk [47, 63]. Nonetheless, DI-GM scores reflect a composite inflammatory profile and may not capture all diet-outcome pathways; thus, context-specific interpretation remains warranted [64, 65].

In this study, we also found that the protective effect of DI-GM was more pronounced in people without CKD, with a higher level of education, and individuals who consume alcohol. We suggest that alcohol may benefit health by modulating gut microbiota. Studies show moderate red wine polyphenol intake beneficially modulates gut microbiota (increasing Bifidobacterium and Bacteroides) [66], reduces cardiovascular risk markers (blood pressure, lipids, CRP) [67], and exerts anti-inflammatory effects *via* antioxidant pathways and intestinal regulatory T cell (Treg) promotion [68, 69]. Our finding aligns with the research by Chen and colleagues [70], who examined the association between inflammatory scores and mortality in patients with metabolic syndrome. Their study also highlighted the central role of inflammatory pathways when it comes to predicting outcomes in people with metabolic diseases. Additionally, a study conducted by Pammer and team noted that among patients with CKD [71], the blood glucose and lipid-related components of metabolic syndrome are the key factors driving both death and cardiovascular events. This insight further reinforces why the DII and the CRP-to- CDAI were so important in the mediation analysis we carried out in our own study.

In this study, we observed that the highest-performing group, Q4, did not differ significantly from Q1, which suggests a potential threshold effect: the marginal benefit of a high-quality diet may plateau beyond a certain level. Accordingly, a continuous DI-GM analysis with marginal-effect curves is warranted to evaluate linearity *versus* nonlinearity in the dose-response relationship. Potential confounders, including medication use, socioeconomic status, comorbidity severity, and drinking patterns, appeared to cluster within the Q4 region and could bias the results; we should further assess these factors in sensitivity analyses and within a propensity-score framework. Additionally, we detected a stronger association among drinkers, which necessitates discussion of the underlying biological mechanisms, gut barrier integrity, inflammation, and metabolic pathways. Future studies should incorporate related biomarkers to enhance the interpretability of these associations.

To our knowledge, this is the first study to systematically evaluate the prognostic value of the DI-GM in adults with CKM syndrome, using a large, nationally representative sample with linked mortality follow-up to improve generalizability. By integrating multivariable-adjusted models, restricted cubic spline analyses, mediation analyses, and subgroup analyses, we characterized associations between DI-GM and mortality risk. We also examined the combined association of DI-GM and the DII, providing additional insight into the diet-microbiome-inflammation axis in CKM progression. 

## 5 STUDY LIMITATIIONS

There are several limitations to these strengths as well. In particular, among older adults, 24-hour dietary recalls are susceptible to recall bias, within-person variability, and measurement error. In the analysis, primarily non-Hispanic Whites were included, limiting generalizability to other races and ethnicities. As an observational study, despite extensive multivariable adjustment, residual confounding cannot be excluded, and causal inferences cannot be established.

## CONCLUSION

In analyses of 7,291 adults with CKM syndrome using linked mortality follow-up, a moderate DI-GM score (5-7) was associated with lower all-cause mortality, and this association appeared to be partially mediated by anti-inflammatory and antioxidant pathways. Based on our findings, DI-GM appears to be a useful tool for nutritional assessment and risk stratification in CKM, with significant associations particularly among participants without CKD, with a higher educational attainment, and who reported alcohol consumption. Randomized clinical trials are needed to test DI-GM-guided personalized nutrition interventions and to evaluate microbiota remodeling strategies in advanced CKD to address the therapeutic challenge of immunometabolic uncoupling. Such studies could strengthen the evidence base for gut microbiota-targeted nutritional interventions for CKM syndrome.

## Figures and Tables

**Fig. (1):**
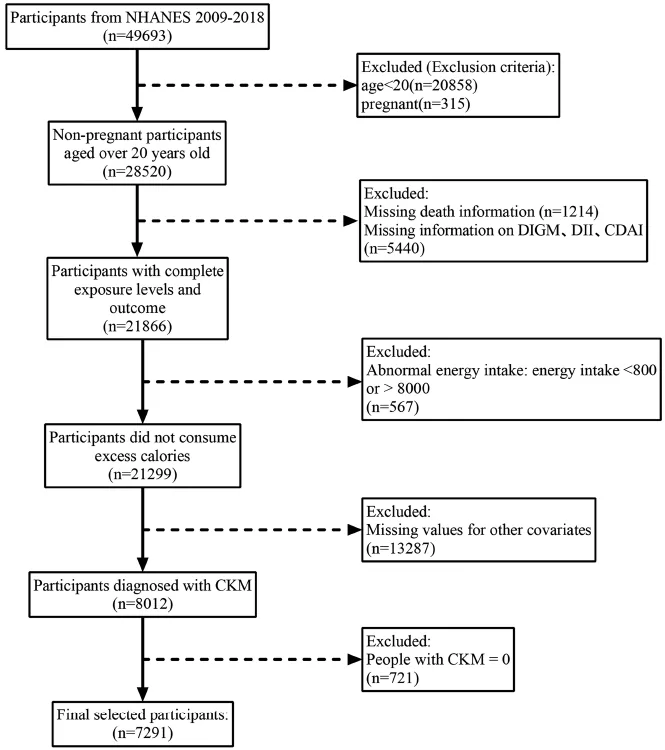
Flow chart of sample selection.

**Fig. (2):**
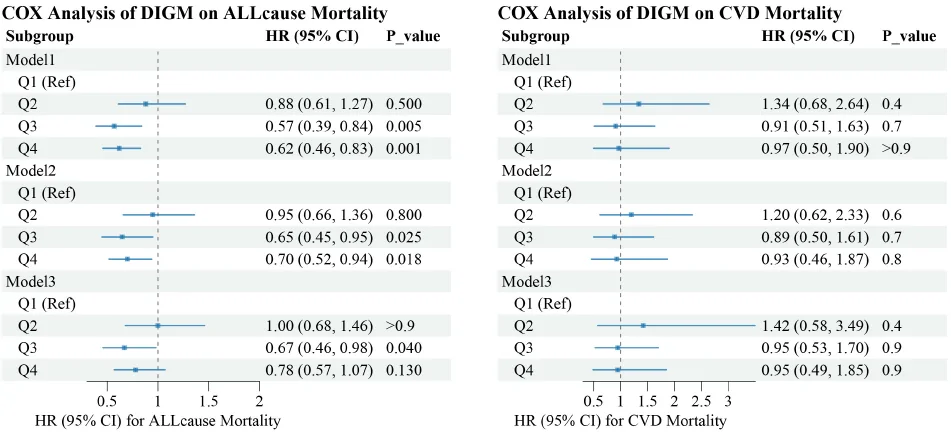
Cox Proportional Hazards Models for the Association Between Dietary Index for Gut Microbiota (DIGM) and All-Cause or Cardiovascular Disease (CVD) Mortality. Model 1: Adjusted for age, sex, and race/ethnicity. Model 2: Model 1 plus socioeconomic status (education, poverty-income ratio), lifestyle factors (body mass index, smoking status, alcohol use). Model 3: Model 2 plus clinical comorbidities (hypertension, diabetes, chronic kidney disease, malignancy, metabolic syndrome) and dietary covariates (total energy intake, dietary cholesterol intake). Q1 is reference. HR, hazard ratio; CI, confidence interval.

**Fig. (3):**
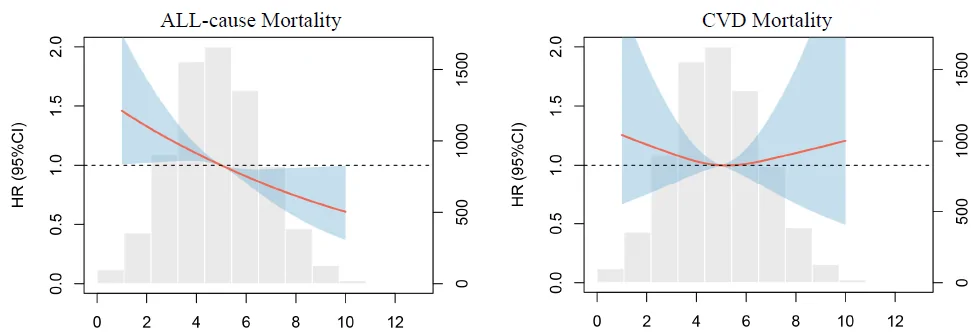
Restricted Cubic Spline Analyses of Dietary Index for Gut Microbiota (DIGM) on All-Cause and Cardiovascular Disease (CVD) Mortality. The model was adjusted for age, gender, and race, with the reference point (HR = 1) set at the median DIGM score. Four knots were placed at the 5th, 35th, 65th, and 95th percentiles of the DIGM distribution.

**Fig. (4):**
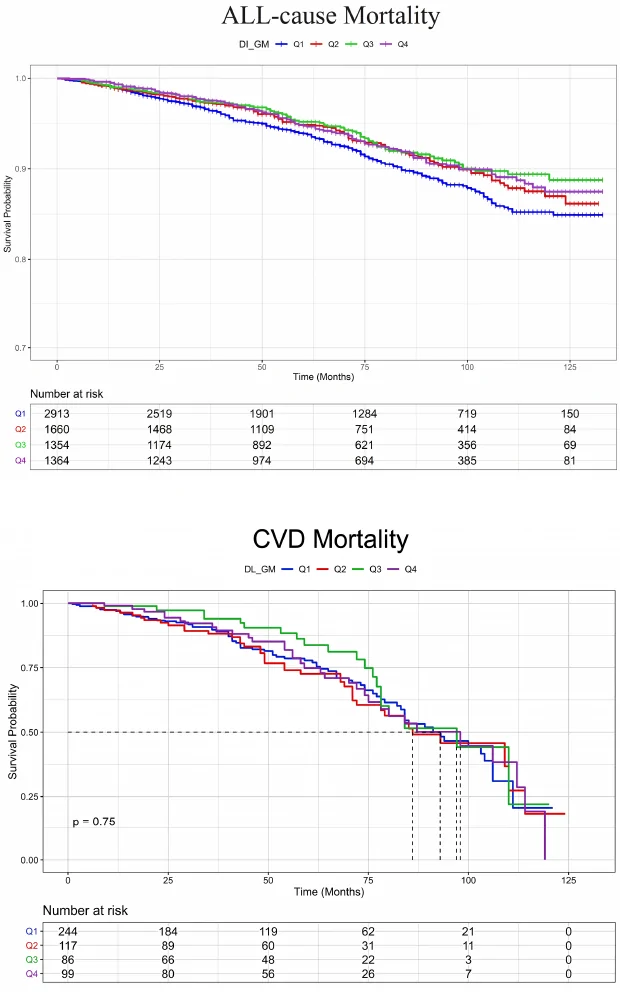
Kaplan-Meier Analyses of Dietary Index for Gut Microbiota (DIGM) on All-Cause and Cardiovascular Disease (CVD) Mortality. The model was adjusted for age, gender, and race. Survival curves and number at risk are presented. Higher DIGM quartiles (Q3-Q4) correlate with better all-cause mortality survival, while no significant difference is observed for CVD mortality (*p*= 0.75). Q1 is the reference group.

**Fig. (5):**
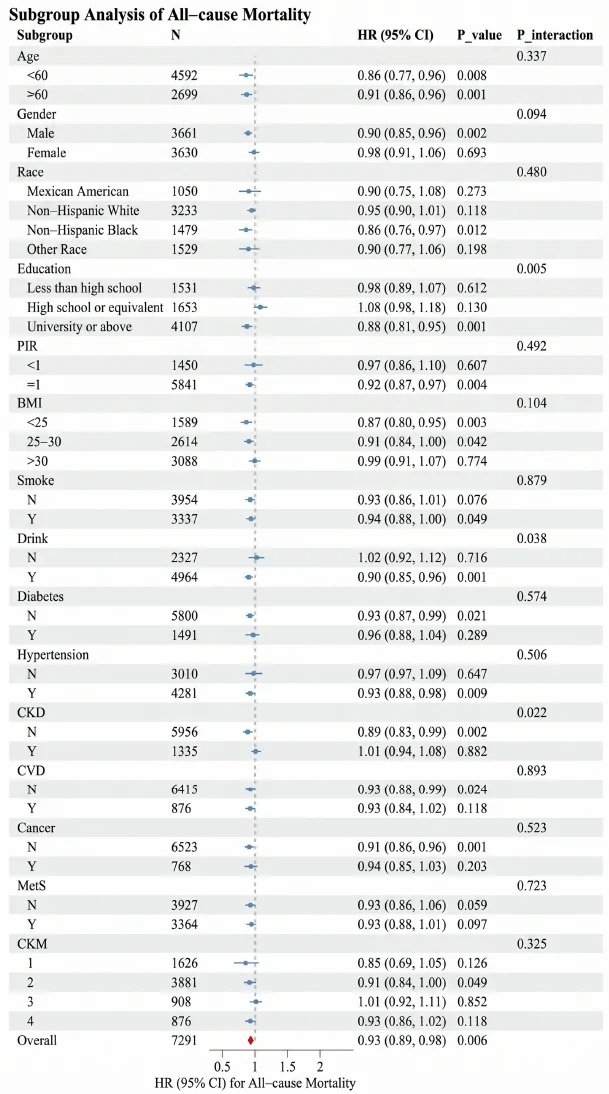
Subgroup Analyses of the Association Between Dietary Index for Gut Microbiota (DIGM) and All-Cause Mortality. HRs (95% CI), *p*-values, interaction *p*-values. DIGM reduces all-cause mortality in subgroups (*e.g*., females, non-Hispanic White, diabetes, CKD; *p* < 0.05) with no significant subgroup interactions (*p* > 0.05).

**Fig. (6):**
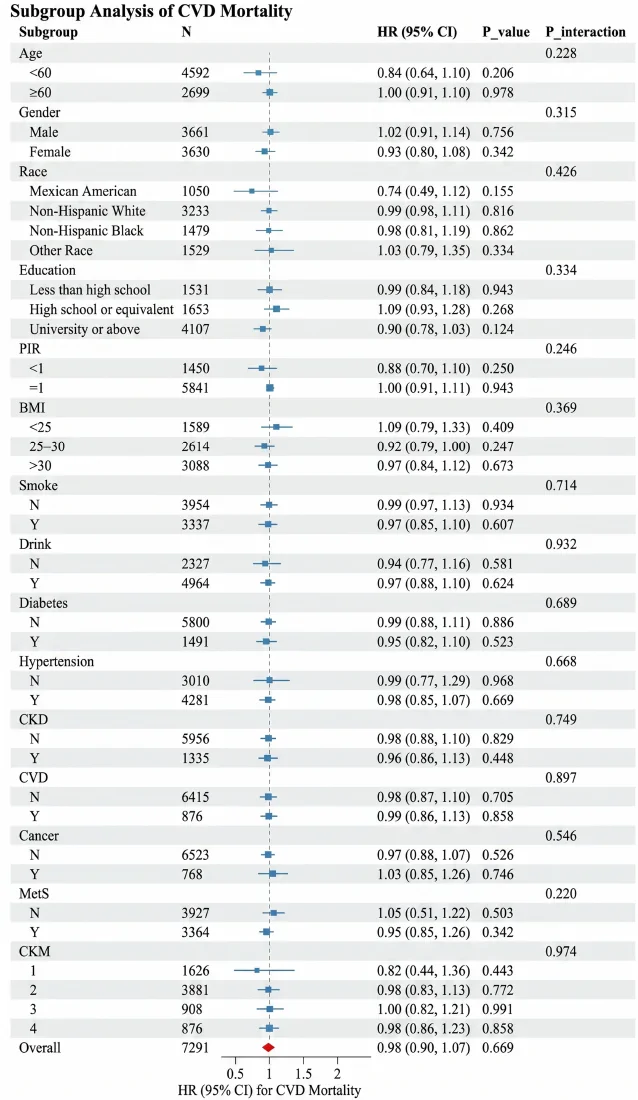
Subgroup Analyses of the Association Between Dietary Index for Gut Microbiota (DIGM) and Cardiovascular Disease (CVD) Mortality. HRs (95% CI), *p*-values, interaction P-values. No significant associations or subgroup interactions for DIGM and CVD mortality (*p*> 0.05).

**Fig. (7):**
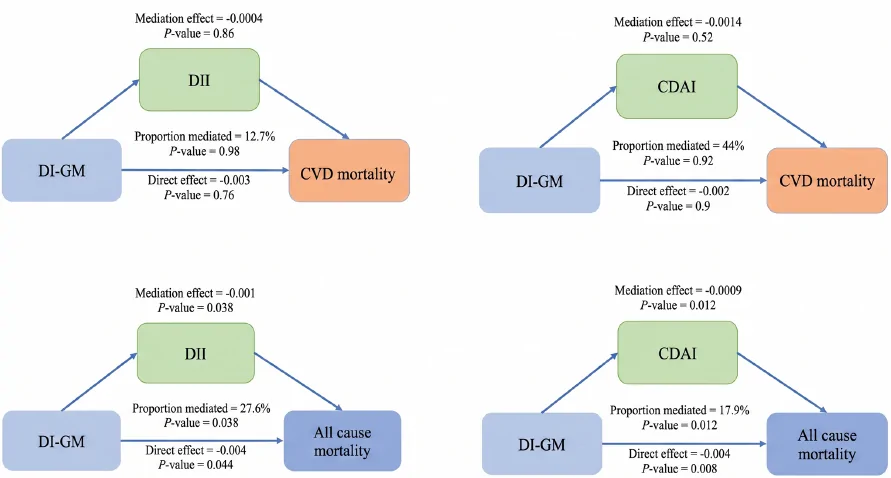
Mediation Analyses of DI-GM on CVD Mortality and All-Cause Mortality *via* Dietary Inflammatory Index (DII) and Composite Dietary Antioxidant Index (CDAI). Pathways depict mediation effects, proportion mediated, direct effects, and corresponding *p*-values. DII and CDAI partially mediate the associations of DI-GM with all-cause mortality (statistically significant for all-cause mortality pathways, *p* < 0.05), while their mediation effects on CVD mortality lack significance (*p* > 0.05).

**Table 1:** The characteristics of patients with CKM syndrome stage 1-4 concerning all-cause mortality and cardiovascular mortality, weighted for representativeness.

-			**CKM**				-
**Characteristic**	**N** ^1^	**Overall**, N = 7291 (100%)^2^	**CKM=1** N = 1626 (26%)^2^	**CKM=2 ** N = 3881 (54%)^2^	**CKM=3 ** N = 908 (10%)^2^	**CKM=4** N = 876 (10%)^2^	***p* Value** ^3^
Age, years	7,291	51.0 (36.0, 63.0)	38.0 (28.0, 51.0)	51.0 (38.0, 60.0)	64.0 (50.0, 74.0)	66.0 (58.0, 74.0)	<0.001
Gender, %	7,291	-	-	-	-	-	0.005
Male	-	3,661 (51%)	728 (48%)	2,022 (53%)	405 (44%)	506 (53%)	-
Female	-	3,630 (49%)	898 (52%)	1,859 (47%)	503 (56%)	370 (47%)	-
Race, %	7,291	-	-	-	-	-	0.004
Mexican American	-	1,050 (8.6%)	268 (10.0%)	584 (8.7%)	126 (8.2%)	72 (4.9%)	-
Non-Hispanic White	-	3,233 (68%)	694 (66%)	1,626 (66%)	420 (69%)	493 (75%)	-
Non-Hispanic Black	-	1,479 (10%)	302 (9.9%)	787 (10%)	200 (11%)	190 (11%)	-
Other Race	-	1,529 (14%)	362 (14%)	884 (15%)	162 (12%)	121 (9.3%)	-
Education Level, %	7,291	-	-	-	-	-	<0.001
Less than high school	-	1,531 (14%)	275 (11%)	806 (14%)	207 (15%)	243 (22%)	-
High school or equivalent	-	1,653 (23%)	307 (20%)	866 (23%)	243 (28%)	237 (25%)	-
University or above	-	4,107 (63%)	1,044 (68%)	2,209 (63%)	458 (57%)	396 (53%)	-
Family Poverty Income Ratio, %	7,291	2.94 (1.44, 5.00)	3.22 (1.63, 5.00)	3.01 (1.42, 5.00)	2.59 (1.35, 4.56)	2.31 (1.33, 4.28)	0.002
BMI, kg/m²	7,291	29 (25, 34)	27 (25, 31)	29 (26, 34)	30 (26, 35)	29 (25, 35)	<0.001
Waist, cm	7,291	100 (91, 111)	95 (88, 103)	102 (93, 113)	105 (94, 116)	104 (94, 118)	<0.001
Smoke, %	7,291	-	-	-	-	-	<0.001
N	-	3,954 (54%)	1,015 (60%)	2,095 (54%)	494 (54%)	350 (39%)	-
Y	-	3,337 (46%)	611 (40%)	1,786 (46%)	414 (46%)	526 (61%)	-
Drink, %	7,291	-	-	-	-	-	<0.001
N	-	2,327 (28%)	443 (25%)	1,210 (27%)	365 (39%)	309 (30%)	-
Y	-	4,964 (72%)	1,183 (75%)	2,671 (73%)	543 (61%)	567 (70%)	-
FBG, mg/dL	7,291	101 (95, 110)	98 (92, 104)	102 (96, 111)	107 (99, 128)	107 (97, 124)	<0.001
GH, %	7,291	5.50 (5.20, 5.80)	5.40 (5.10, 5.60)	5.50 (5.30, 5.80)	5.80 (5.40, 6.40)	5.80 (5.48, 6.30)	<0.001
Urea Nitrogen, mg/dL	7,291	13.0 (11.0, 16.0)	13.0 (10.0, 15.0)	13.0 (11.0, 16.0)	16.0 (12.0, 21.0)	16.0 (12.0, 20.4)	<0.001
Creatinine, mg/dL	7,291	0.85 (0.72, 0.99)	0.83 (0.71, 0.95)	0.84 (0.71, 0.97)	0.95 (0.74, 1.19)	0.96 (0.81, 1.14)	<0.001
Uric Acid, mg/dL	7,291	5.50 (4.60, 6.40)	5.03 (4.30, 5.80)	5.60 (4.70, 6.50)	5.90 (5.00, 7.10)	5.80 (4.90, 6.90)	<0.001
Triglyceride, mg/dL	7,291	110 (77, 159)	81 (61, 103)	135 (87, 182)	123 (86, 182)	116 (87, 176)	<0.001
Cholesterol , mg/dL	7,291	190 (164, 217)	184 (161, 209)	196 (172, 225)	189 (165, 224)	171 (144, 201)	<0.001
HDL, mg/dL	7,291	51 (43, 62)	55 (48, 66)	49 (41, 59)	51 (42, 62)	49 (41, 62)	<0.001
LDL, mg/dL	7,291	112 (91, 137)	111 (91, 130)	117 (96, 142)	110 (90, 139)	92 (74, 120)	<0.001
Caloric Intake, Kcal	7,291	2,004 (1,580, 2,569)	2,083 (1,646, 2,639)	2,032 (1,594, 2,590)	1,766 (1,424, 2,289)	1,870 (1,467, 2,412)	<0.001
Fat Intake, mg/day	7,291	77 (58, 103)	80 (60, 108)	78 (58, 102)	70 (52, 91)	73 (54, 99)	<0.001
Cholesterol Intake, mg/day	7,291	253 (168, 379)	253 (167, 379)	256 (172, 382)	249 (166, 380)	247 (162, 358)	0.6
DIGM	7,291	5.00 (4.00, 6.00)	5.00 (4.00, 6.00)	5.00 (4.00, 6.00)	5.00 (4.00, 6.00)	5.00 (4.00, 6.00)	0.080
CDAI	7,291	0.5 (-1.5, 3.0)	1.0 (-1.2, 3.5)	0.5 (-1.5, 2.9)	-0.1 (-1.9, 2.6)	0.2 (-1.8, 2.2)	<0.001
DII	7,291	1.35 (-0.12, 2.62)	1.08 (-0.33, 2.50)	1.38 (-0.05, 2.57)	1.73 (0.10, 2.90)	1.55 (0.25, 2.80)	0.001
Hypertension, %	7,291	-	-	-	-	-	<0.001
N	-	3,010 (46%)	1,626 (100%)	1,071 (29%)	177 (21%)	136 (18%)	-
Y	-	4,281 (54%)	0 (0%)	2,810 (71%)	731 (79%)	740 (82%)	-
Diabetes, %	7,291	-	-	-	-	-	<0.001
N	-	5,800 (85%)	1,626 (100%)	3,094 (84%)	565 (65%)	515 (67%)	-
Y	-	1,491 (15%)	0 (0%)	787 (16%)	343 (35%)	361 (33%)	-
CKD, %	7,291	-	-	-	-	-	<0.001
N	-	5,956 (85%)	1,626 (100%)	3,833 (99%)	0 (0%)	497 (62%)	-
Y	-	1,335 (15%)	0 (0%)	48 (1.3%)	908 (100%)	379 (38%)	-
MetS, %	7,291	-	-	-	-	-	<0.001
N	-	3,927 (56%)	1,626 (100%)	1,660 (42%)	326 (36%)	315 (40%)	-
Y	-	3,364 (44%)	0 (0%)	2,221 (58%)	582 (64%)	561 (60%)	-
CVD, %	7,291	-	-	-	-	-	<0.001
N	-	6,415 (90%)	1,626 (100%)	3,881 (100%)	908 (100%)	0 (0%)	-
Y	-	876 (10%)	0 (0%)	0 (0%)	0 (0%)	876 (100%)	-
Cancer, %	7,291	-	-	-	-	-	<0.001
N	-	6,523 (89%)	1,544 (94%)	3,535 (90%)	760 (85%)	684 (78%)	-
Y	-	768 (11%)	82 (6.0%)	346 (9.7%)	148 (15%)	192 (22%)	-
All cause- mortality, %	7,291	546 (5.6%)	31 (1.4%)	170 (3.8%)	152 (14%)	193 (17%)	<0.001
CVD- mortality, %	546	166 (29%)	7 (17%)	48 (26%)	34 (27%)	77 (35%)	0.4
Follow-up Time, Month	7,291	68 (38, 97)	72 (39, 100)	69 (38, 98)	59 (35, 91)	61 (34, 93)	0.013

^1^N not Missing (unweighted);^ 2^median (IQR) for continuous; n (%) for categorical; ^3^Wilcoxon rank-sum test for complex survey samples; chi-squared test with Rao & Scott's second-order correction

## Data Availability

The data sets used and/or analysed during this study are available from the corresponding author upon request.

## LIST OF ABBREVIATIONS

ACME: Average Causal Mediation Effect
BMI: Body Mass Index
CKD: Chronic kidney disease
CKM Syndrome: Cardiovascular-Kidney-Metabolic Syndrome
CDAI: Composite Dietary Antioxidant Index
CVD: Cardiovascular Disease
DI-GM: Dietary Index for Gut Microbiota
DII: Dietary Inflammatory Index
HR: Hazard Ratio
NHANES: National Health and Nutrition Examination Survey
PIR: Family Income-to-Poverty Ratio
RCS: Restricted Cubic Splines
VIF: Variance Inflation Factor
