# Supplementary material for: Dietary Index for Gut Microbiota and All-Cause Mortality in CKM Syndrome: Evidence of a J-Shaped Association and Partial Mediation by DII and CDAI
Source: Endocr Metab Immune Disord Drug Targets. 2026 Jun 10;26:E18715303459900. doi: 10.2174/0118715303459900260505102519 (PMC13635967; doi:10.2174/0118715303459900260505102519)
Supplement: Supplementary file 1 [file EMIDDT-26-E18715303459900_SD1.pdf]

Supplementary Material

Dietary Index for Gut Microbiota and All-Cause Mortality in CKM Syndrome: Evidence of a J Shaped Association and Partial Mediation by DII and CDAI

Xiaoqi Ma[1,#], Jingwen Zhang[1,#], Jinjin Huang[1], Ting Liu[2], Jingai Fang[2] and Xiaodong Zhang[2,*]

[1]*Department of Nephrology, Shanxi Medical University, First School of Clinical Medicine, Taiyuan, China;* [2]*Department of Nephrology, The First Hospital of Shanxi Medical University, Taiyuan, China*

Supplementary Table S1. Definitions of CKM.

CKM conditions	Definition	CKM indicators	Threshold for CKM indicators
CVD	Individuals with clinical CVD or subclinical CVD	Clinical CVD	History of chronic heart failure, coronary heart disease, heart attack, or stroke
		Subclinical CVD	Any of the following criterion is met:<br><br>1) Very high-risk CKD in KDIGO classification: UACR ≥ 300 mg/g and eGFR ≤ 45-59 ml/min/1.73m2, UACR ≥ 30 mg/g and eGFR ≤ 30-44 ml/min/1.73m2, or eGFR ≤ 29 ml/min/1.73m2.<br><br>2) Predicted 10-year CVD risk ≥ 20%
Kidney diseases	Individuals with CKD	CKD	Moderate-to-high-risk CKD in KDIGO classification: UACR ≥ 30 mg/g and eGFR ≥ 60 ml/min/1.73m2, UACR < 300 mg/g and eGFR ≤ 45-59 ml/min/1.73m2, or UACR < 30 mg/g and eGFR ≤ 30-44 ml/min/1.73m2.
Metabolic disorders	Individuals<br><br>With overweight/obesity, abdominal obesity, prediabetes,<br><br>diabetes,<br><br>hypertension, hypertriglyceridemia<br><br>or MetS	Overweight/obesity	BMI ≥25 kg/m2 (or ≥23 kg/m2 if Asian ancestry)*
		Abdominal obesity	Waist circumference ≥88/102 cm in female/male (or if Asian ancestry ≥80/90 cm in female/male)
		Prediabetes	Fasting blood glucose ≥ 100-124 mg/dL or HbA1c ≥ 5.7%-6.4% and without self-reported diagnosis of diabetes, use of insulin, or oral hypoglycemic agents
		Diabetes	Fasting blood glucose ≥ 125 mg/dL or HbA1c ≥ 6.5% or self-reported diagnosis of diabetes, use of insulin, or oral hypoglycemic agents
		Hypertension	SBP ≥130 mm Hg or DBP ≥80 mm Hg or self-reported diagnosis of hypertension or use of antihypertensive medications
		Hypertriglyceridemia	Triglycerides ≥ 135 mg/dL
		MetS	MetS is defined by the presence of 3 or more of the following:<br><br>1)Waist circumference ≥88/102 cm in female/male (or if Asian ancestry ≥80/90 cm in female/male).<br><br>2) HDL cholesterol <50/40 mg/dL in female/male.

			3)Triglycerides ≥150 mg/dL.<br><br>4)Elevated blood pressure (SBP ≥130mmHg or DBP ≥80 mmHg and/or use of anti-hypertensive medications).<br><br>5)Fasting blood glucose ≥100 mg/dL

*Asian was not listed as a separate race/ethnicity until NAHNES 2011-2012, therefore the uniform threshold for BMI and waist circumference was used in all participants in NHANES 1999-2010.

Abbreviations: BMI, body mass index; CKD, chronic kidney disease; CKM, cardiovascular-kidney-metabolic syndrom; CVD, cardiovascular disease; DBP, diastolic blood pressure; eGFR, estimated glomerular filtration rate; HDL-C, high-density lipoprotein cholesterol; KDIGO, The Kidney Disease: Improving Global Outcomes; MetS, metabolic syndrome; SBP, systolic blood pressure; UACR, urinary albumin to creatinine ratio.

Supplementary Table S2. Detailed algorithm for evaluating each CKM stage.

CKM stages	Definition	Criterion	Threshold for CKM conditions
Stage 0: No CKM risk factors	Individuals with normal BMI and waist circumference, normoglycemia, normotension, a normal lipid profile, and no evidence of CKD or subclinical or clinical CVD	All criteria are met	BMI <25 kg/m^2 (or <23 kg/m^2 if Asian ancestry)*
			Waist circumference <88/102 cm in female/male (or if Asian ancestry <80/90 cm in female/male)
			Fasting blood glucose < 100 mg/dL and HbA1c < 5.7% and without self-reported diagnosis of diabetes, use of insulin, or oral hypoglycemic agents
			SBP <130 mm Hg and DBP <80 mm Hg without self-reported diagnosis of hypertension or use of antihypertensive medications
			HDL cholesterol >50/40 mg/dL in female/male and triglycerides < 150 mg/dL
			Low-risk CKD in KDIGO classification according to eGFR and UACR: UACR < 30 mg/g and eGFR ≥ 60 ml/min/1.73m^2.
			Predicted 10-year CVD risk < 20%
			No clinical CVD
Stage 1: Excess or dysfunctional adiposity	Individuals with overweight/obesity, abdominal obesity, or dysfunctional adipose tissue, without the presence of other metabolic risk factors or CKD	Any of the three criteria is met	Overweight/obesity
			Abdominal obesity
			Prediabetes
		All criteria are met	SBP <130 mm Hg and DBP <80 mm Hg without self-reported diagnosis of hypertension or use of antihypertensive medications
			HDL cholesterol >50/40 mg/dL in female/male and triglycerides <150 mg/dL

			Low-risk CKD in KDIGO classification according to eGFR and UACR: UACR < 30 mg/g and eGFR ≥ 60 ml/min/1.73m^2
			Predicted 10-year CVD risk < 20%
			No clinical CVD
Stage 2: Metabolic risk factors and CKD	Individuals with metabolic risk factors (hypertriglyceridemia, hypertension, MetS, diabetes), or CKD	Any of the five criteria is met	Hypertriglyceridemia
			Hypertension
			diabetes
			MetS
			Moderate-to-high-risk CKD in KDIGO classification
		All criteria are met	No very high-risk CKD in KDIGO classification
			Predicted 10-year CVD risk < 20%
			No clinical CVD
Stage 3: Subclinical CVD in CKM	Subclinical CVD among individuals with excess/dysfunctional adiposity, other metabolic risk factors, or CKD	Any of the two criteria is met	Very high-risk CKD in KDIGO classification
			Predicted 10-year CVD risk ≥ 20%
		Any of the eight criteria is met	Overweight/obesity
			Abdominal obesity
			Prediabetes
			Hypertriglyceridemia
			Hypertension
			diabetes
			MetS
			Moderate-to-high-risk CKD in KDIGO classification
		The criterion is met	No clinical CVD
Stage 4: Clinical CVD in CKM	Clinical CVD among individuals with excess/dysfunctional adiposity, other metabolic risk factors, or CKD	The criterion is met	Clinical CVD
		Any of the nine criteria is met	Overweight/obesity
			Abdominal obesity
			Prediabetes
			Hypertriglyceridemia

			Hypertension
			diabetes
			MetS
			Moderate-to-high-risk CKD in KDIGO classification
			Very high-risk CKD in KDIGO classification

*Asian was not listed as a separate race/ethnicity until NHANES 2011-2012, therefore the uniform threshold for BMI and waist circumference was used in all participants in NHANES 1999-2010.

Among the study participants, 77 individuals had missing values for the 10-year CVD risk score. However, these participants could still be clearly classified as stage 3 or 4 of cardio-vascular-kidney-metabolic syndrom based on other available variables.

Abbreviations: BMI, body mass index; CKD, chronic kidney disease; CKM, cardiovascular-kidney-metabolic syndrom; CVD, cardiovascular disease; DBP, diastolic blood pressure; eGFR, estimated glomerular filtration rate; HDL-C, high-density lipoprotein; KDIGO, The Kidney Disease: Improving Global Outcomes; NHANES, National Health and Nutrition Examination Survey; SBP, systolic blood pressure; UACR, urinary albumin to creatinine ratio.

Supplementary Table S3. Components and scoring criteria of DI-GM in NHANES.

**Components of DI-GM**	**Food Items Included in NHANES**	**Scoring Criteria**
**Beneficial to gut microbiota**	Avocados	Score 1 - Consumption≥sex-specific median<br><br>Score 0 - Otherwise
	Broccoli	
	Chickpeas	
	Coffee	
	Cranberries	
	Fermented dairy (including yogurt, cheese, kefir, sour cream, buttermilk)	
	Fiber	
	Soybean (including Soy milk, Tofu)	
	Whole grains	
**Unfavorable to gut microbiota**	Refined grains	Score 0 - Consumption≥sex-specific median<br><br>Score 1 - Otherwise
	Processed meat	
	Red meat	
	High-fat diet (% energy)	Score 0 - Consumption≥40%<br><br>Score 1 - Otherwise

Supplementary Table S4. Variance Inflation Factor (VIF) values obtained from the fitted collinearity diagnostics.

Variable	VIF_1	VIF_2	VIF_3
DI_GM	1.246815	1.229745	1.292715
CDAI	3.228045	3.210758	4.645754
DII	3.296682	3.230011	3.773464
Age	2.00927	1.864037	1.559663
Gender	2.122634	1.974344	1.821092
Race	1.210457	1.20176	1.131606
Education	1.483925	1.465566	1.336376
PIR	1.380332	1.375225	1.37314
BMI	7.245967	1.408221	1.473447
WAIST	7.49578	-	-
Smoke	1.165753	1.158162	1.275674
drink	1.183565	1.182315	1.356368
GLU	3.43425	3.493953	3.296125
GH	3.540337	3.548446	3.333378
SBU	1.790163	1.776858	2.433192
SCR	1.757309	1.75387	1.856327
SUA	1.569972	1.566806	1.492824
TR	6.23697	1.786947	1.718972
CH	49.58902	1.417679	1.614642
HDD	8.637091	1.855672	1.788758
LDL	38.34683	-	-
ACT	1.149479	1.149199	1.254551
Hypertension	1.31461	1.514485	1.249226
Diabetes	1.957915	1.9604	1.903431
Cancer	1.109169	1.107311	1.093244
KCAL	6.095354	2.216971	2.6614
FAT	5.339165	-	-
CHOL	1.661236	1.557537	1.960607
CKD	1.375686	1.374718	1.5035
CVD	1.218608	1.216616	1.20067

If all VIF values are below 5, multicollinearity among the variables in the model is considered low and the model is stable. If any variable has a VIF greater than 10, that variable should be removed or combined with others to reduce collinearity. VIF-1 includes all variables. VIF-2 shows the linear association between all-cause mortality and the remaining variables after removing waist, dietary fat, and LDL. VIF-3 shows the linear association between cardiovascular mortality and the remaining variables after removing waist, dietary fat, and LDL.

Supplementary Table S5. Associations of DIGM with All-cause mortality、CVD mortality and Other mortality in CKM.

DIGM	ALL-cause mortality		CVD mortality	
	HR（95%Cl）	p-value	HR（95%Cl）	p-value
**Model1**				
Q1	Ref		Ref	
Q2	0.88（0.61，1.27）	0.5	1.34（0.68，2.64）	0.4
Q3	0.57（0.39，0.84）	0.005	0.91（0.51，1.63）	0.7
Q4	0.62（0.46，0.83）	0.001	0.97（0.50，1.90）	> 0.9
**Model2**				
Q1	Ref		Ref	
Q2	0.95（0.66，1.36）	0.8	1.20（0.62，2.33）	0.6
Q3	0.65（0.45，0.95）	0.025	0.89（0.50，1.61）	0.7
Q4	0.70（0.52，0.94）	0.018	0.93（0.46，1.87）	0.8
**Model3**				
Q1	Ref		Ref	
Q2	1.00（0.68，1.46）	> 0.9	1.42（0.58，3.49）	0.4
Q3	0.67（0.46，0.98）	0.04	0.95（0.53，1.70）	0.9
Q4	0.78（0.57，1.07）	0.13	0.95（0.49，1.85）	0.9

HR = Hazard Ratio, CI = Confidence Interval

Supplementary Table S6. Subgroup analyses for the association between quartile of DIGM and all-cause mortality、CVD mortality.

Variable	Count	ALL cause mortality			CVD mortality		
		HR (95%Cl)	P value	P for interaction	HR (95%Cl)	P value	P for interaction
Age				0.337			0.228
<60	4592	0.86 (0.77,0.96)	0.008		0.84 (0.64,1.1)	0.206	
>= 60	2699	0.91 (0/86,0.96)	0.001		1 (0.91,1.1)	0.978	
Gender				0.094			0.315
Male	3661	0.9 (0.85,0.96)	0.002		1.02 (0.91,1.14)	0.756	

Female	3630	0.98 (0.91,1.06)	0.693		0.93 (0.8,1.08)	0.342	
Race				0.48			0.426
Mexican American	1050	0.9 (0.75,1.08)	0.273		0.74 (0.49,1.12)	0.155	
Non-Hispanic White	3233	0.95 (0.9,1.01)	0.118		0.99 (0.88,1.11)	0.816	
Non-Hispanic Black	1479	0.86 (0.76,0.97)	0.012		0.98 (0.81,1.19)	0.862	
Other Race	1529	0.9 (0.77,1.06)	0.198		1.03 (0.79,1.35)	0.834	
Education				0.005			0.334
Less than high school	1531	0.98 (0.89,1.07)	0.612		0.99 (0.84,1.18)	0.943	
High school or equivalent	1653	1.08 (0.98,1.18)	0.13		1.09 (0.93,1.28)	0.268	
University or above	4107	0.88 (0.81,0.95)	0.001		0.9 (0.78,1.03)	0.124	
PIR				0.492			0.246
<1	1450	0.97 (0.86,1.1)	0.607		0.88 (0.7,1.1)	0.25	
>= 1	5841	0.92 (0.87,0.97)	0.004		1 (0.91,1.11)	0.943	
BMI				0.104			0.369
<25	1589	0.87 (0.8,0.95)	0.003		1.09 (0.89,1.33)	0.409	
>30	3088	0.99 (0.91,1.07)	0.774		0.97 (0.84,1.12)	0.673	
25-30	2614	0.91 (0.84,1)	0.042		0.92 (0.79,1.06)	0.247	
Smoke				0.879			0.714
N	3954	0.93 (0.86,1.01)	0.076		0.99 (0.87,1.13)	0.934	
Y	3337	0.94 (0.88,1)	0.049		0.97 (0.85,1.1)	0.607	
Drink				0.038			0.932
N	2327	1.02 (0.92,1.12)	0.716		0.94 (0.77,1.16)	0.581	
Y	4964	0.9 (0.85,0.96)	0.001		0.97 (0.88,1.08)	0.624	
Diabetes				0.574			0.689
N	5800	0.93 (0.87,0.99)	0.021		0.99 (0.88,1.11)	0.886	
Y	1491	0.96 (0.88,1.04)	0.289		0.95 (0.82,1.1)	0.523	
Hypertension				0.506			0.668
N	3010	0.97 (0.87,1.09)	0.647		0.99 (0.77,1.29)	0.968	
Y	4281	0.93 (0.88,0.98)	0.009		0.98 (0.89,1.08)	0.669	
CKD				0.022			0.749
N	5956	0.89 (0.83,0.96)	0.002		0.98 (0.86,1.13)	0.829	
Y	1335	1.01 (0.94,1.08)	0.882		0.96 (0.85,1.07)	0.448	
CVD				0.893			0.897
N	6415	0.93 (0.88,0.99)	0.024		0.98 (0.87,1.1)	0.705	

Y	876	0.93 (0.86,1.02)	0.118		0.99 (0.86,1.13)	0.858	
Cancer				0.523			0.546
N	6523	0.91 (0.86,0.96)	0.001		0.97 (0.88,1.07)	0.526	
Y	768	0.94 (0.85,1.03)	0.203		1.03 (0.85,1.26)	0.746	
MetS				0.723			0.22
N	3927	0.93 (0.86,1)	0.059		1.05 (0.91,1.22)	0.503	
Y	3364	0.95 (0.88,1.01)	0.097		0.95 (0.85,1.06)	0.342	
CKM				0.325			0.974
1	1626	0.85 (0.69,1.05)	0.126		0.82 (0.49,1.36)	0.443	
2	3881	0.91 (0.84,1)	0.049		0.98 (0.83,1.15)	0.772	
3	908	1.01 (0.92,1.11)	0.852		1 (0.82,1.21)	0.991	
4	876	0.93 (0.86,1.02)	0.118		0.99 (0.86,1.13)	0.858	
Overall	7291	0.93 (0.89,0.98)	0.006		0.98 (0.9,1.07)	0.669	

Supplementary Table S7. Mediation analysis of diet indices on cardiovascular and all-cause mortality.

Type		CDAI				DII			
		Estimate	CI_Lower	CI_Upper	P_value	Estimate	CI_Lower	CI_Upper	P_value
CVD mortality									
	ACME	-0.0014	-0.0066	0.0029	0.522	-0.0004	-0.008	0.0068	0.864
	ADE	-0.0018	-0.0245	0.0227	0.898	-0.0029	-0.0256	0.0228	0.834
	Total	-0.0033	-0.0253	0.0207	0.76	-0.0033	-0.0253	0.0207	0.76
	PropMed	0.4403	-3.2277	4.2418	0.922	0.1267	-3.7974	4.9629	0.984
All-cause mortality									
	ACME	-0.0009	-0.0016	-0.0002	0.012	-0.0014	-0.0027	-0.0001	0.038
	ADE	-0.0042	-0.0075	-0.0007	0.008	-0.0037	-0.0072	-0.0001	0.044
	Total	-0.0052	-0.0084	-0.0016	0	-0.0052	-0.0084	-0.0016	0
	PropMed	0.1795	0.036	0.5998	0.012	0.2762	0.0188	0.9644	0.038

Supplementary Table S8. Association of DIGM with All-cause mortality and CVD mortality in patients with CKM.

DIGM	ALL-cause mortality		CVD mortality	
	HR（95%Cl）	*p*-value	HR（95%Cl）	*p*-value
Model1	0.91 (0.84, 0.98)	0.012	0.99 (0.86, 1.13)	0.9
Model2	0.93 (0.87, 1.0)	0.035	1.0 (0.87, 1.13)	＞0.9
Model3	0.96 (0.90, 1.03)	0.2	1.01 (0.90, 1.14)	0.8

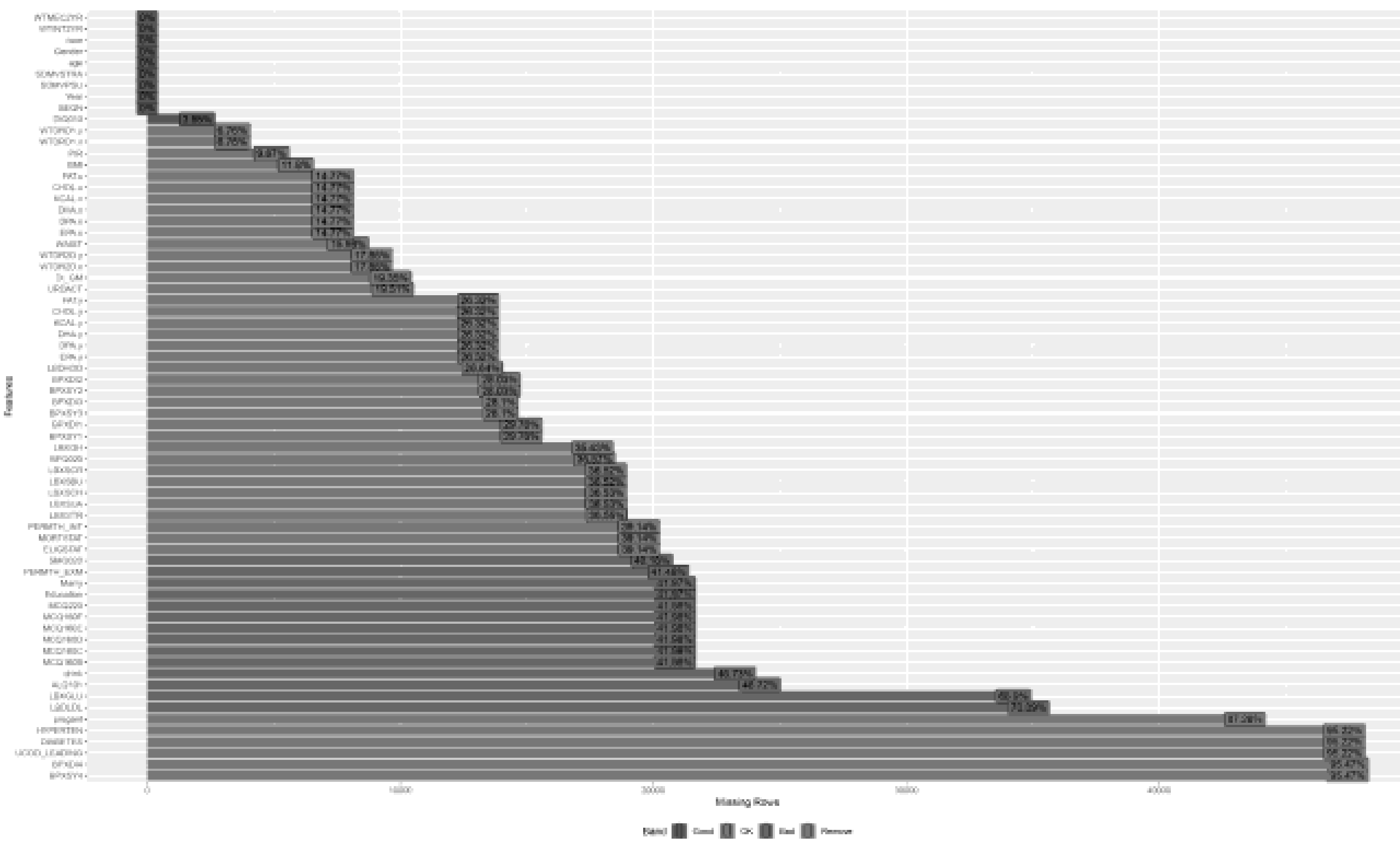

Supplementary Figure S1. Original data visualization summary.

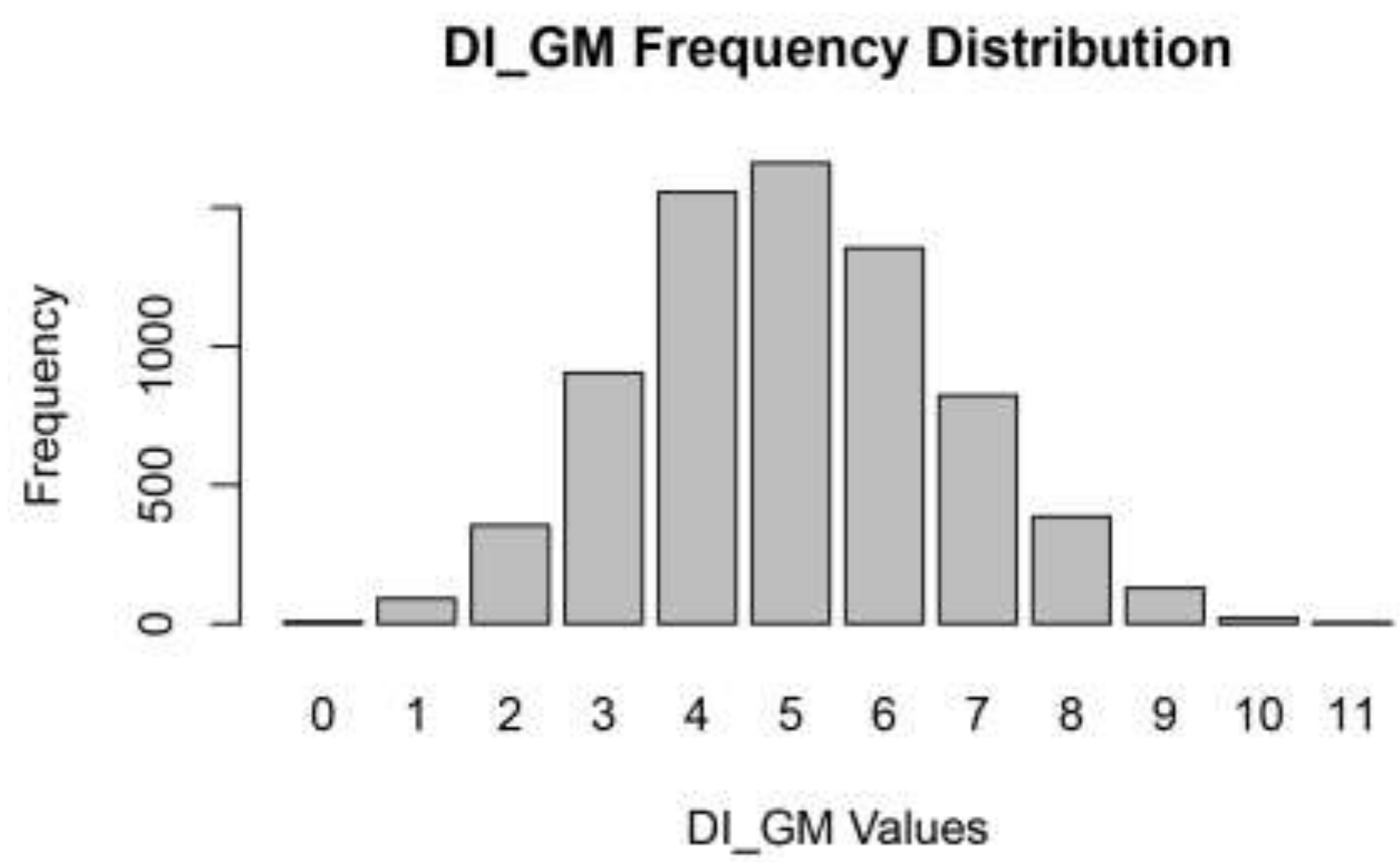

Supplementary Figure S2. DIGM Data visualization summary.
